# BiVO_4_ Ceramic Photoanode with Enhanced Photoelectrochemical Stability

**DOI:** 10.3390/nano11092404

**Published:** 2021-09-15

**Authors:** Liren Zheng, Minrui Wang, Yujie Li, Fahao Ma, Jiyu Li, Weiyi Jiang, Mu Liu, Hefeng Cheng, Zeyan Wang, Zhaoke Zheng, Peng Wang, Yuanyuan Liu, Ying Dai, Baibiao Huang

**Affiliations:** 1 State Key Laboratory of Crystal Materials, Shandong University, Jinan 250100, China; 201720279@mail.sdu.edu.cn (L.Z.); 201611976@mail.sdu.edu.cn (M.W.); 18306422339@163.com (Y.L.); mafahao@mail.sdu.edu.cn (F.M.); jiyuli1226@gmail.com (J.L.); jiangweiyi1994@mail.sdu.edu.cn (W.J.); liumu@mail.sdu.edu.cn (M.L.); chenghefeng@sdu.edu.cn (H.C.); zkzheng@sdu.edu.cn (Z.Z.); pengwangicm@sdu.edu.cn (P.W.); yyliu@sdu.edu.cn (Y.L.); 2 School of Physics and Electronic Engineering, TaiShan University, Tai’an 271000, China; 3 School of Physics, Shandong University, Jinan 250100, China; daiy60@sina.com

**Keywords:** BiVO_4_ photoanode, photoelectrochemical stability, spark plasma sintering (SPS), ceramics

## Abstract

Monoclinic bismuth vanadate (BiVO_4_) is an attractive material with which to fabricate photoanodes due to its suitable band structure and excellent photoelectrochemical (PEC) performance. However, the poor PEC stability originating from its severe photo-corrosion greatly restricts its practical applications. In this paper, pristine and Mo doped BiVO_4_ ceramics were prepared using the spark plasma sintering (SPS) method, and their photoelectrochemical properties as photoanodes were investigated. The as-prepared 1% Mo doped BiVO_4_ ceramic (Mo-BVO (C)) photoanode exhibited enhanced PEC stability compared to 1% Mo doped BiVO_4_ films on fluorine doped Tin Oxide (FTO) coated glass substrates (Mo-BVO). Mo-BVO (C) exhibited a photocurrent density of 0.54 mA/cm^2^ and remained stable for 10 h at 1.23 V vs. reversible hydrogen electrode (RHE), while the photocurrent density of the Mo-BVO decreased from 0.66 mA/cm^2^ to 0.11 mA/cm^2^ at 1.23 V vs. RHE in 4 h. The experimental results indicated that the enhanced PEC stability of the Mo-BVO (C) could be attributed to its higher crystallinity, which could effectively inhibit the dissociation of vanadium in BiVO_4_ during the PEC process. This work may illustrate a novel ceramic design for the improvement of the stability of BiVO_4_ photoanodes, and might provide a general strategy for the improvement of the PEC stability of metal oxide photoanodes.

## 1. Introduction

Photoelectrochemical (PEC) water splitting is regarded as one of the most high-potential strategies to solve environmental and energy issues for human society in the future can, as it can produce hydrogen and oxygen by directly utilizing solar energy. To withstand the conditions of oxidation and maintain long-term stability in aqueous solution, oxide semiconductors, such as TiO_2_ [1], ZnO [2], BiVO_4_ [3], and WO_3_ [4], etc, have become the preferred photoelectrode candidates. Among these materials, BiVO_4_ has received great attention as a photoanode for photoelectrochemical water splitting owing to its suitable band structure and excellent semiconductor properties [5,6]. Consequently, BiVO_4_ is an ideal photoanode and enables oxygen evolution at a low bias, in contrast to many other metal oxides [7,8]. The maximum photocurrent of BiVO_4_ photoanode could be up to 7.5 mA/cm^2^ under AM1.5 G light irradiation, with a theoretical conversion efficiency of over 9% [9]. To improve the photoelectrochemical performance of BiVO_4_ photoanodes, great efforts have been made by researchers all over the world, such as improving the light absorption of the materials by optimizing their morphology and surface structure [10,11,12], doping with transition metal ions to improve electrical conductivity [13,14], loading coa catalyst on the surface of the electrode in order to accelerate water oxidation kinetics [7,14], and constructing a heterojunction to stimulate the transportation and separation of photogenerated electrons and holes [15,16].

Although the photocurrent of BiVO_4_ photoanodes has been greatly improved, their stability has not been well addressed. According to research, the poor photoelectrochemical (PEC) stability of BiVO_4_ photoanodes could be attributed to the dissociation of V into the solution from the electrode, which can be greatly accelerated under light illumination, resulting in more severe photo-corrosion [17,18,19]. Depositing a protective layer, or the modification of a layer of cocatalysts on the surface of the electrode is the most commonly used method for the prevention of surface photo-corrosion and for the improvement of the stability of BiVO_4_ photoanodes [7,14]. Recently, it was found that the photo-corrosion and the instability of photoanodes were also closely related to surface defects. Our previous investigations found that the PEC stability of single-crystalline ZnO photoanodes with fewer surface defects was much higher than that of ZnO photoanodes consisting of ZnO nanorods [2]. Thus, if we could fabricate a BiVO_4_ photoanode with enhanced crystallinity, its PEC stability might also be improved. Additionally, with increased crystallinity, there would be fewer surface defects on the BiVO_4_ photoanode, which could subsequently reduce the recombination of photogenerated electrons and holes on the electrode’s surface. This could also further improve the PEC performance of BiVO_4_ photoanodes [20,21]. However, BiVO_4_ single crystals are quite difficult and complex to produce in comparison with the simple fabrication procedure of BiVO_4_ polycrystalline films. In this regard, BiVO_4_ ceramics fabricated by BiVO_4_ powders could offer an alternative to BiVO_4_ single crystals, which not only exhibit higher crystallinity than BiVO_4_ films consisting of nanoparticles, but might also be easier to fabricate than BiVO_4_ single crystals.

Based on this idea, BiVO_4_ ceramics photoanodes with (Mo-BVO (C)) or without (BVO (C)) Mo doping were prepared by using the spark plasma sintering (SPS) method [22]. For comparison, we prepared the Mo doped BiVO_4_ (Mo-BVO) films on FTO substrate. The photocurrent of the Mo-BVO (C) electrode was 0.54 mA/cm^2^ and that of the Mo-BVO film was 0.66 mA/cm^2^ under 1.23 V (vs. reversible hydrogen electrode (RHE)). After a long-term water splitting reaction, the Mo-BVO (C) showed better stability than the Mo-BVO electrode under identical conditions. The Mo-BVO (C) exhibited a photocurrent density of 0.54 mA/cm^2^ and remained stable for 10 h at 1.23 V vs. RHE. By contrast, the photocurrent density of the Mo-BVO decreased from 0.66 mA/cm^2^ to 0.11 mA/cm^2^ at 1.23 V vs. RHE in 4 h. By comparing the morphologies, structures, and surface states of the as-prepared Mo-BVO (C) and the Mo-BVO, we attributed the excellent PEC stability of the Mo-BVO (C) to its lower number of surface defects and itshigher crystallinity, which could inhibit the dissociation of V in BiVO4 and suppress the photo-corrosion during PEC process. Therefore, the preparation of ultra-thin BiVO_4_ materials with high density and high crystallization is a rational strategy to effectively improve the PEC stability of BiVO_4_ semiconductor.

## 2. Experimental Details

### 2.1. Raw Materials

Bismuth (III) nitrate (Bi(NO_3_)_3_·5H_2_O), ammonium metavanadate (NH_4_VO_3_), vanadium acetylacetonate (VO(acac)_2_), bis(acetylacetonato)dioxomolybdenum (MoO_2_(acac)_2_), and hexaammonium molybdate ((NH_4_)_6_Mo_7_O_24_·4H_2_O) were purchased from shanghai Aladdin Biochemical Technology Co., Ltd, Shanghai, China. Citric acid, Nitric acid (69 wt%), and liquid ammonia (30 wt%) were obtained from Sinopharm Chemical Reagent Co Ltd., Shanghai, China. All the other chemicals were of analytical grade and were used as received without further purification. All the solutions were prepared with deionized (DI) water obtained from a PURE ROUP 30 water purification system.

### 2.2. Preparation of BiVO_4_ and Mo doped BiVO_4_ Powders

The BiVO_4_ and Mo doped BiVO_4_ powders were synthesized using a modified sol-gel route [23,24]. In a typical synthetic procedure, 40 mmol Bi(NO_3_)_3_·5H_2_O were dissolved into 200 mL of 3 M nitric acid under stirring, then 24 g of citric acid and 60 mL of deionized water were added, respectively. The mixture was magnetically stirred for 30 min. The pH of the above mixture was adjusted to 7.00 with an ammonia solution through vigorous stirring at room temperature, and the solution A was obtained. The solution B contained ammonium metavanadate (NH_4_VO_3_) (4.633 g) and citric acid (24 g) in 200 mL boiling water. The pH of the solution B was adjusted to 7.00 by controlling the addition of NH_4_OH, or nitric acid. To prepare the bismuth vanadate precursor solution, A and B solutions were mixed together with the Bi: V molar ratio of 1.0:1.0. To obtain the Mo doped BiVO_4_, a certain amount of molybdenum source ((NH_4_)_6_Mo_7_O_24_) was added to solution B. Next, the A and B solutions were mixed together, with a Bi: V: Mo molar ratio of 1:0.99:0.01, in order to obtain the 1% Mo doped BiVO_4_ precursor. Subsequently, the mixture solution was put into an oil bath pot at 80 °C with magnetic stirring. After 10–15 h, the homogeneous blue sol was formed, transferred to an oven, and maintained at 100 °C for 4 days, until it became a dark, brown, dry gel. Following this, the gel was grounded into powder by an agate mortar and pestle. Next, the powders were calcined in a muffle furnace at 500 °C for 4 h. Finally, when the temperature was cooled to room temperature, the bright yellow powder was collected to make the BiVO_4_ ceramics.

### 2.3. Preparation of BiVO_4_ and Mo Doped BiVO_4_ Ceramics

10 g of BiVO_4_ (or Mo-BiVO_4_) powders were placed in a graphite mold. Next, the mold was placed in the sintering furnace of plasma at 66 MPa of uniaxial pressure, the door of the furnace chamber was closed, and the vacuum pump was opened. At a pressure of 66 MPa, a heating rate of 100 °C/min was kept at 700 °C for 10 min. Next, the temperature was cooled down to room temperature and the ceramic sample was taken out of the chamber. The coated graphite paper was then removed with sandpaper. The density of the ceramic was measured according to the Archimedes principle. The sintering time of ceramics should not be too long. At high temperatures (above 450 °C), BiVO_4_ would decompose, which would result in the loss of vanadium via volatile VO_x_, i.e., VO_2_, species according to research [25].

### 2.4. Fabrication of BiVO_4_ and Mo Doped BiVO_4_ Films on FTO Substrate

The 1% Mo doped BiVO_4_ photoanode was prepared with the spin-coating method and then calcined at a high temperature [26,27]. FTO glass cleaned by ethanol, acetone, deionized water, and glycol was used as the substrate. In a specific preparation process, 0.72 mmol of Bi(NO_3_)_3_·5H_2_O, 0.633 g of vanadium acetylacetonate, and bis(acetylacetonato)dioxomolybdenum (0.0048 g) were dissolved in glacial acetic acid and acetylacetone, respectively. Subsequently, the two solutions were evenly mixed and magnetically stirred for 20 min, and then the precursor solution was obtained by ultrasound at room temperature for 6 h. The precursor was spin-coated onto FTO glass at 700 rpm for 10 s. Next, the FTO substrate was dried at 150 °C for 10 min, and then calcined at 450 °C for 30 min. The above process was repeated four times, before a photoanode with a thickness of 215 nm was obtained. The FTO was cut into many small strips (size: 0.5 × 1.0 cm^2^) with a diamond glass knife; the solution area was 0.25 mm^2^. The preparation of pure BiVO_4_ film (BVO) followed the same method and procedure as that of the Mo doped BiVO_4_ film, except for the addition of bis(acetylacetonato)dioxomolybdenum in the precursor solution.

### 2.5. Preparation of BiVO_4_ and Mo-BiVO_4_ Ceramic Photoanode

The BVO and Mo-BVO ceramics ware cut into many square flakes (5.0 × 5.0 × 0.3 mm^3^) with a diamond wire cutting machine. Next, the two sides were polished with sandpaper and alumina slurry. The thickness of the BVO (C) and the Mo-BVO (C) was optimized around 10–30 μm. Next, one side of the ceramic chip was coated in Gallium-Indium eutectic and treated at 500 °C for 20 min in a muff furnace to obtain Ohmic contact. Subsequently, copper wires were connected to the surface of the Gallium-Indium eutectic with silver paste. When the silver glue was completely dry, the metal surface and copper wire were sealed with epoxy resin to prevent electricity leakage. The area of the photoelectrode was about 1–3 mm^2^.

### 2.6. Photoelectrochemical Characterizations

The photoelectrochemical properties of these BiVO_4_ photoanodes were studied in a three-electrode configuration using the as-prepared BiVO_4_ ceramic or film photoelectrode as the working electrode, a platinum sheet as the counter electrode, and a standard saturated calomel electrode (SEC) as the reference electrode in 0.1 M of potassium phosphate buffer solution (KPi, pH = 7). The data were recorded on an electrochemical workstation (Shanghai Chenhua electrochemical analyzer / CHI600E workstation, Shanghai, China). All light sources were simulated sunlight, which was provided by a 300 W Xe arc lamp equipped with an AM 1.5 G filter (Perfectlight, Beijing, Co. LTD, Beijing, China, 100 mW /cm^2^). The bias voltage of the electrode was converted to a reversible hydrogen electrode (RHE) by the following equation:

$$E_{RHE}=E_{SCE}+0.591pH+0.241$$

The photocurrent density-bias curves (J-V) were taken at a scan rate of 20 mV·s^−1^ in the three-electrode cell of a KPi electrolyte under AM 1.5 G irradiation. The incident-photon-to-current-conversion efficiency (IPCE) measurements were performed by measuring the photocurrent density under monochromated light irradiation with a 500 W Xe arc lamp coupled into a grating monochromator. Electrochemical impedance spectroscopy (EIS) was performed in potentiostatic mode at 0 V bias voltage vs. the reversible hydrogen electrode (RHE) over frequencies ranging from 100 kHz to 100 mHz.

### 2.7. Characterizations

The morphology, crystal structure, and components of the samples as prepared were analyzed by scanning electron microscopy (SEM, a Hitachi S-4800 microscope from Japan with an accelerating voltage of 5 kV), and energy dispersive spectroscopy (EDS) from Tokyo, Japan, X-ray diffraction (XRD, Bruker AXS D8 advance powder diffracto-meter with a Cu Kα X-ray tube, Karlsruhe, Germany). The X-ray photoelectron spectroscopy (XPS) measurements were performed on an ESCALAB 250 photoelectron spectrometer from Massachusetts, US, employing Al Kα radiation (E = 1486.6 eV). The C 1s line located at 284.6 eV was used as the calibration position for all the element data. The diffuse reflectance spectra were measured by using a Shimadzu UV 2550 UV-vis spectrometer from Kyoto, Japan equipped with an integrating sphere in the wavelength range of 200–800 nm. The Raman spectra were obtained by excitation of the samples with a 473 nm laser on a LabRAM HR800 Raman spectrometer (Horiba Jobin Yvon) from Paris, France.

## 3. Results and Discussion

### 3.1. Morphologies and Structures of As-Prepared Samples

The scanning electron microscopy (SEM) images of the BiVO_4_ powder precursors used to fabricate the BVO (C) and the Mo-BVO (C) are shown in Figure S1. The BiVO_4_ precursor powders with and without Mo doping obtained by sol-gel methods exhibited similar morphologies, which were spherical or ellipsoidal, with sizes of 0.4~1.5 μm, and consisted of nanoparticles of 100~200 nm. The surface area of the doped and undoped powders measured by nitrogen adsorption and desorption were also quite similar: 2.77 m^2^/g and 2.82 m^2^/g, respectively.

**Figure 1:**
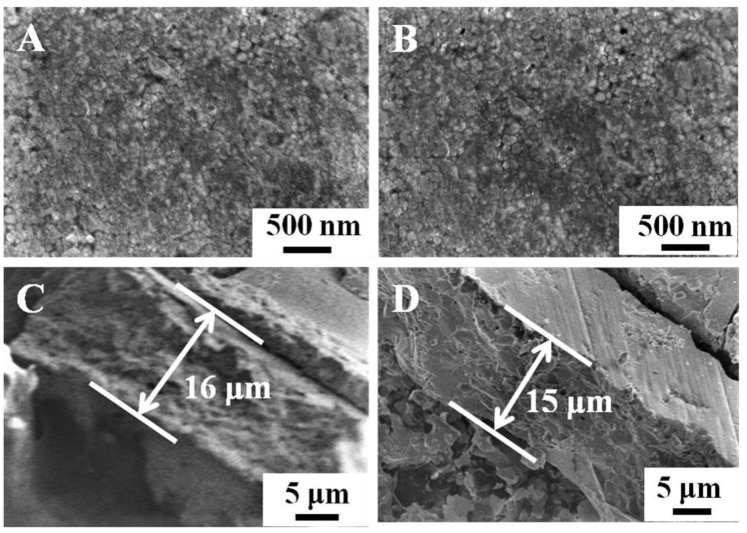
(**A**,**B**) show SEM images of the BVO (C) and Mo-BVO (C) ceramics fabricated by the BiVO_4_ precursor powders. (**C**,**D**) show cross-sectional images of the as-prepared BVO (C) and Mo-BVO (C).

Figure 1 shows the surfaces and the cross-section of the BVO (C) and Mo-BVO (C) ceramics. A few bubbles or holes can be observed on the surface of the ceramics chips, indicating that the as-prepared BVO (C) and Mo-BVO (C) showed high densities. The densities of the as-prepared BVO (C) and Mo-BVO (C) were measured to be 98.5% and 99.8% of the theoretical density for monoclinic BiVO_4_ (6.9 g/cm^3^) [28], respectively. The grain sizes of both ceramic samples were about 1~3 μm. The cross-sectional image shown in Figure 1C,D indicates that the thickness of the as-prepared BVO (C) and Mo-BVO (C) was about 15 μm. Although the thickness of the as-prepared BVO (C) and Mo-BVO (C) ceramics were still much larger than the diffusion length of the photogenerated electrons, which could lower the bulk charge separation efficiency during the PEC process, the ceramic chips would have easily broken during the electrode fabrication process if we had made them thinner. For comparison, the SEM images of the BVO and Mo-BVO films on FTO substrates are also presented, as shown in Figure S2. The morphologies of the BVO and Mo-BVO films are quite different from the BVO (C) and Mo-BVO (C), as shown in Figure 1, which mainly consisted of inter-connected nanoparticles with sizes of 10–50 nm. There were numerous voids between these nanoparticles. Furthermore, the thickness of both the BVO and the Mo-BVO films was about 200 nm.

### 3.2. X-ray Diffraction and UV-Vis DRS Measurements

The XRD patterns of the BVO (C) and Mo-BVO (C), shown in Figure S3, can be identified as monoclinic BiVO_4_ (JPCDS No. 14-688) without any other impurity peaks. For the BVO and Mo-BVO, the XRD peaks corresponding to the FTO substrates (marked by * in Figure S3) can be observed beside the XRD peaks corresponding to the monoclinic BiVO_4_.

**Figure 2:**
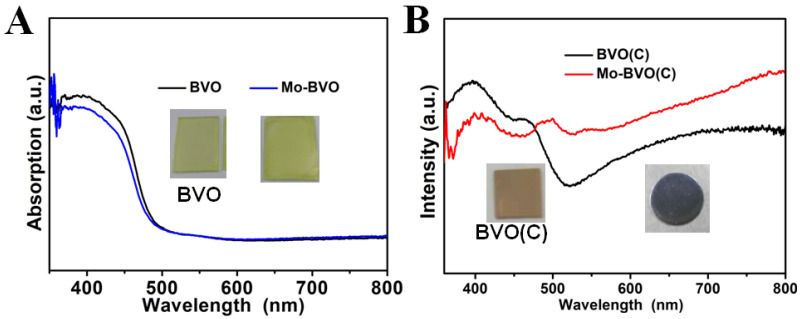
(**A**) UV-vis DRS of BVO and Mo-BVO. (**B**) UV-vis DRS of BVO (C) and Mo-BVO(C). The inset shows the digital photos of BVO, Mo-BVO, BVO(C), and Mo-BVO(C).

The light absorption of the as-prepared BVO and Mo-BVO films on the FTO substrates and the BVO (C) and Mo-BVO (C) ceramics were characterized by UV-Vis diffused reflectance spectra. As shown in Figure 2B, the BVO (C) exhibited a pale-yellow color with an absorption edge of 517 nm. However, after Mo doping, the Mo-BVO (C) exhibited a dark grey color with light absorption in the whole visible region. This is quite similar to the case of Mo doped BiVO_4_ single crystals reported in a different study [29]. According to this study, the change of sample color after Mo doping could be ascribed to the formation of shallow donor impurities, which can give rise to a low-energy transition between impurity levels and the conduction band of BiVO_4_. As a result, all visible photons can be absorbed by Mo doped BiVO_4_ ceramics or single crystals. This dark grey color is thought to be closely related to the size of the crystal domains, where the dark grey color of the Mo-BVO (C) turns back to yellow after grinding, as illustrated in Figure S4. Both the BVO and Mo-BVO films on the FTO substrates exhibited similar light absorption curves with a light absorption edge at 500 nm, as shown in Figure 2A.

### 3.3. Photoelectrochemical Properties of BVO, BVO(C), Mo-BVO(C), and Mo-BVO

**Figure 3:**
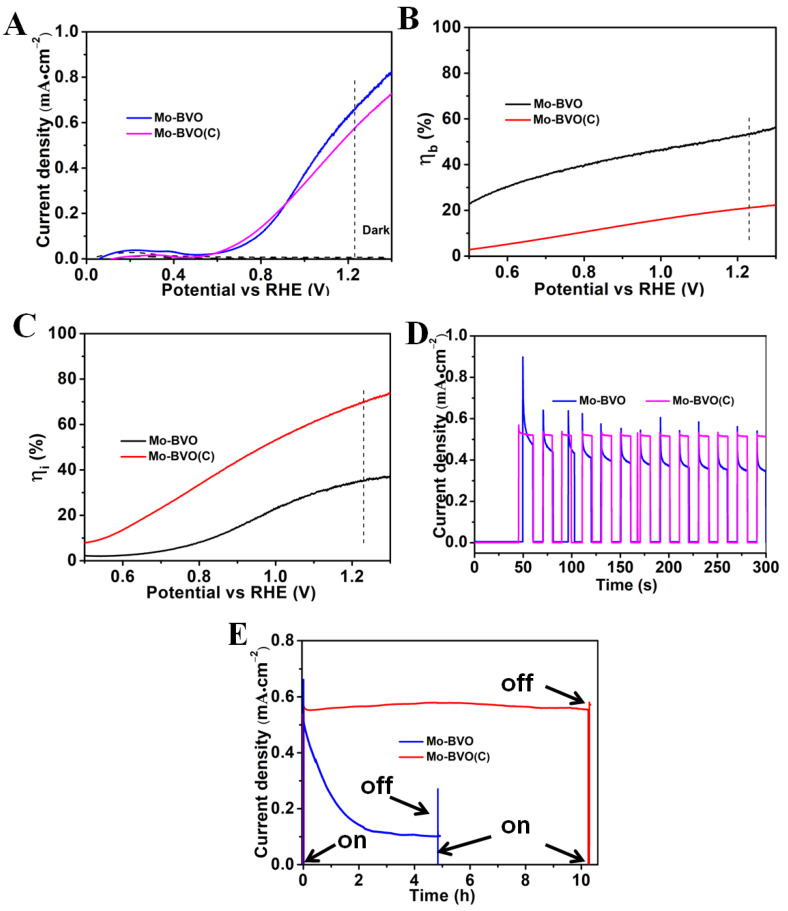
(**A**) Photocurrent density vs. applied potential (J−V) curves measured for the Mo-BVO and Mo-BVO (C) photoanodes, with a scan rate of 20 mV/s under AM 1.5 G illumination in 0.1 M KPi buffer solution. (**B**) The bulk charge separation efficiencies ($\eta_{b}$) and (**C**) interfacial charge separation efficiencies ($\eta_{i}$) of Mo-BVO and Mo-BVO (C) as a function of applied bias, respectively. (**D**) Photocurrent vs. time (J−T) measurements performed for the Mo-BVO and Mo-BVO (C) photoanodes with chopped light (AM 1.5 G) at 1.23 V (vs. RHE) in a phosphate buffer (pH = 7) solution. (**E**) J−T curves of the Mo-BVO and the Mo-BVO (C) measured at 1.23V (vs. RHE) for 10 h in a KPi solution (PH = 7).

The photocurrent density versus applied potential (J-V) and the photocurrent density versus time curves (J-T) of the BVO(C) and the BVO are shown in Figure S5. Figure 3 shows the PEC performances of the Mo-BVO and Mo-BVO (C) samples. The photocurrents of the Mo-BVO (C) and Mo-BVO photoanodes were quite similar under AM1.5 G simulated solar light irradiation, as shown in Figure 3A. At 1.23 V vs. RHE, the photocurrent densities for the Mo-BVO and the Mo-BVO (C) were 0.54 and 0.66 mA/cm^2^, respectively. The lower photocurrent density of the Mo-BVO (C) was due to the larger thickness of the ceramic chips compared to those in the Mo-BVO films, which lowered the bulk charge separation efficiency as the photogenerated electrons transported across the photoanode. To prove this point, the bulk ($\eta_{b}$) and interfacial ($\eta_{i}$) charge separation efficiency of the as-prepared Mo-BVO and Mo-BVO (C) were calculated following the procedure shown in SI. The $\eta_{b}$ and $\eta_{i}$ of the two electrodes vs. the bias voltage are shown in Figure 3B,C. As shown in Figure 3B, the $\eta_{b}$ of the Mo-BVO and the Mo-BVO (C) at 1.23 V vs. RHE were 53.4% and 22.0%, respectively. This is in accordance with the analysis mentioned above. The Mo-BVO (C) was as thick as ~15 μm, which is much thicker than Mo-BVO film (~200 nm). Thus, more electrons would have recombined as they transported across the electrode in the Mo-BVO (C) than in the Mo-BVO film, which lead to a lower $\eta_{b}$ value for the Mo-BVO (C). Electrochemical impendence spectra (EIS) analysis were also carried out at 0 V vs. SCE under light irradiation. The Nyquist plots of Mo-BVO (C) and Mo-BVO are presented in Figure S6. As can be seen, the Mo-BVO exhibited a smaller semicircle than the Mo-BVO (C), which indicates that the charge transfer resistance (R_CT_) in the Mo-BVO was smaller than that in the Mo-BVO (C). By fitting the Nyquist plots according to the equivalent circuit, the R_CT_ for the Mo-BVO (C) and the Mo-BVO were estimated at 11.3 and 6.5 kΩ, respectively, which is also accordance with the $\eta_{b}$ values mentioned above.

The $\eta_{i}$ of Mo-BVO (C) was much higher than that of the Mo-BVO film. As shown in Figure 3C, the $\eta_{i}$ values for the Mo-BVO (C) and the Mo-BVO at 1.23 V vs. RHE were 69.6% and 35.3%, respectively. This indicates that there were fewer defects on the surface of the Mo-BVO (C) than on that of the Mo-BVO because the surface defects would have been the main recombination centers for the photogenerated electrons and holes at the electrode/electrolyte interface during the PEC process. Additionally, the stability of the BiVO_4_ photoanodes was also regarded as closely dependent on the number of surface defects; in other words, the higher the surface defects, the lower the stability of the photoanode. Thus, the PEC stability of the as-prepared Mo-BVO (C) was expected to be much better than that of the Mo-BVO film.

In order to evaluate the PEC stability of the as-prepared Mo-BVO (C) and Mo-BVO photoanodes, the steady state photocurrents were measured at 1.23 V vs. RHE under chopped AM 1.5 G simulated solar light. As shown in Figure 3D, the Mo-BVO (C) exhibited better PEC stability, as expected. The photocurrent density of the Mo-BVO (C) was kept at 0.54 mA/cm^2^ without any decay in 300 s, while the photocurrent density of the Mo-BVO rapidly decreased from 0.66 mA/cm^2^ to 0.40 mA/cm^2^ in 300 s. On closer observation of the transient photocurrent pulse, the shapes of the pulse for the Mo-BVO (C) and the Mo-BVO were also quite different. As the light was turned on, the photocurrent of both the Mo-BVO (C) and the Mo-BVO increased rapidly. However, the photocurrent of the Mo-BVO rapidly decreased until the light was off, while the photocurrent of the Mo-BVO (C) slightly decreased and stabilized rapidly. This indicates that the recombination of photogenerated charge carriers in the Mo-BVO was much quicker than in the Mo-BVO (C), which could mainly be ascribed to the larger number of surface defects in the Mo-BVO. To address this point, steady state photocurrent measurements were also performed in the presence of Na_2_SO_3_ as a hole scavenger, as shown in Figure S7. The photocurrent density of both the Mo-BVO (C) and the Mo-BVO increased in the presence of 0.1 M Na_2_SO_3_. At 1.23 V vs. RHE, the photocurrent densities of the Mo-BVO (C) and the Mo-BVO were 0.79 and 1.87 mA/cm^2^, respectively. Furthermore, as shown in the J-T plots in Figure S7B, the photocurrents of both the Mo-BVO (C) and the Mo-BVO remained stable without any decay. More importantly, the shape of the transient photocurrent pulse of the Mo-BVO greatly changed, while the photocurrent rapidly increased as the light was turned on and quickly stabilized. This result indicates that the rapid surface recombination of photogenerated electrons and holes could have been the main reason for the poor PEC stability of the as-prepared Mo-BVO, which would subsequently have lead to the rapid decrease in photocurrent. In order to investigate the long-term PEC stability of the Mo-BVO (C) and the Mo-BVO, steady state photocurrent measurements were performed at 1.23 V vs. RHE under AM 1.5 G simulated solar light irradiation for a longer time. As shown in Figure 3E, the Mo-BVO (C) exhibited excellent PEC stability, while the photocurrent kept stable without any obvious decay after 10 h. However, the photocurrent of the Mo-BVO decreased from 0.66 to 0.11 mA/cm^2^ after 4 h.

The incident photon-to-current conversion efficiencies (IPCEs) of the Mo-BVO (C) and the Mo-BVO were also measured, as shown in Figure S8. The IPCEs of the Mo-BVO were slightly higher than those of the Mo-BVO (C). At 420 nm, the IPCE values for the Mo-BVO and the Mo-BVO (C) were 17% and 12%, respectively. The IPCE plots of the Mo-BVO accorded with the DRS spectra, as shown in Figure 2, where the IPCE value become zero as the wavelengths were longer than 500 nm. However, the IPCE plots of the Mo-BVO (C) were quite different from the DRS spectra, as shown in Figure 2. Although the Mo-BVO (C) absorbed photons atwavelengths longer than 500 nm, these photons could not generate any photocurrent during PEC water splitting.

### 3.4. Photoelectrochemical Properties, SEM, XRD, Raman, XPS, and EDS of the Mo-BVO and Mo-BVO (C) Electrodes after the Stability Test

In order to probe the mechanism underlying the good PEC stability of the as-prepared Mo-BVO (C), systematic investigations were carried out to find changes before and after the long-term PEC experiments. For comparison, the same characterizations of the Mo-BVO films on the FTO substrates were also investigated.

**Figure 4:**
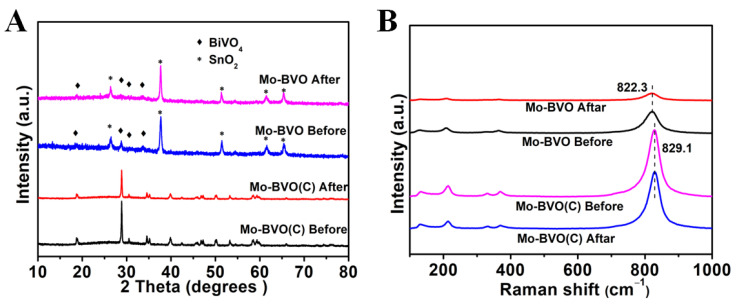
(**A**) XRD data of Mo-BVO and Mo-BVO (C) before and after PEC for 4 h and 10 h in a KPi solution, respectively. (**B**) Raman spectrum of Mo-BVO and Mo-BVO (C) before and after PEC for 4 h and 10 h, respectively.

As shown in Figure 4A, no obvious changes to the structures of Mo-BVO and Mo-BVO (C) were observed after the long-term PEC experiments. Both the Mo-BVO and the Mo-BVO were indexed to monoclinic BiVO_4_ without the appearance of any other impurities. However, the intensity of the XRD of the Mo-BVO was very low, and it becomes lower after four hours of illumination, as shown in Figure 4A. According to the diffraction intensity, the crystallinity of the film was poor. The higher the crystalline quality, the smaller the proportion of defects. The defects acted as trapping and recombination centers for the photogenerated electrons and holes, resulting in a decrease in photoelectrochemical catalytic activity and severe photocorrosion.

The Raman spectra of the two photoelectrodes before and after illumination are shown in Figure 4B. Five Raman peaks were detected in both the Mo-BVO and the Mo-BVO (C), which was ascribed to the characteristic Raman peaks for monoclinic scheelite BiVO_4_. The strongest peaks, at around 822 cm^−1^ (Mo-BVO) and 829 cm^−1^ (Mo-BVO(C)), were ascribed to the antisymmetric stretching modes of the VO_4_ tetrahedra. The bending modes of the VO_4_ tetrahedra at 328 cm^−1^ and 369 cm^−1^, and 323 cm^−1^ and 364 cm^−1^, belonged to the Mo-BVO electrode and Mo-BVO (C) electrode, respectively. The peaks at 126 cm^−1^ and 209 cm^−1^ (Mo-BVO), and 131 cm^−1^ and 214 cm^−1^ (Mo-BVO (C)) were due to the vibration of the crystal lattice [29,30,31]. After long-term PEC experiments, the Raman spectra of Mo-BVO (C) did not change. However, the Raman peaks of the Mo-BVO become weaker and wider after the long-term PEC experiments, as the intensity and the width of the Raman peaks were closely related to the crystallinity of the materials. The change in the Raman peaks for the Mo-BVO before and after the PEC experiments indicated the decrease in the crystallinity of the Mo-BVO.

**Figure 5:**
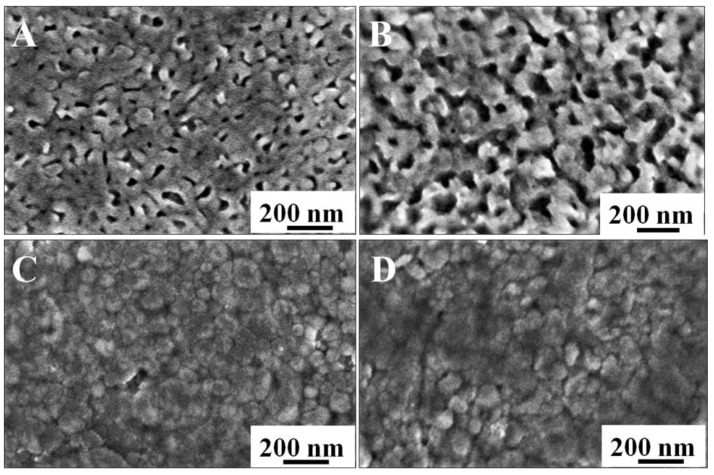
Morphology of Mo-BVO and Mo-BVO (C) before and after 4h and 10 h J-T measurements, respectively. (**A**) Before Mo-BVO. (**B**) After Mo-BVO. (**C**) Before Mo-BVO (C). (**D**) After Mo-BVO (C).

In order to investigate the changes in the morphologies of the Mo-BVO (C) and the Mo-BVO, SEM images for the two samples before and after the PEC stability test were prepared, as shown in Figure 5. As can be seen in Figure 5A,B, great morphological changes were observed for the Mo-BVO before and after the PEC stability test. The original morphology of the Mo-BVO consisted of inter-connected Mo doped BiVO_4_ nanoparticles with smooth surfaces. However, these Mo doped BiVO_4_ nanoparticles were seriously corroded after the PEC experiments at 1.23 V for 4 h. This is consistent with the photocorrosion phenomenon of the reported BiVO_4_ film electrodes [6], in which either the interface between the particles or the surface of particles is corroded preferentially. The case of the Mo-BVO (C) was much better, where the surface morphology did not greatly change before or after the PEC stability test for 10 h.

As the instability of the BiVO_4_ photoanodes was thought to have been due to the partial dissociation of V into the electrolyte solution during the PEC process, the Bi/V ratio before and after the PEC process in the photoanode was expected to increase. To probe the change to the Bi/V ratio, EDS measurements were carried out on the Mo-BVO and the Mo-BVO (C) before and after the PEC stability test. The initial Bi/V ratios for the Mo-BVO (C) and the Mo-BVO were 1: 0.87 and 1:0.80, respectively (see Table S1). However, after the stability test, the Bi/V ratios for the Mo-BVO (C) and the Mo-BVO became 1: 0.73 and 1: 0.73, respectively. This result indicated that more V was dissociated from the Mo-BVO than from Mo-BVO (C). This could explain why the PEC stability of the Mo-BVO (C) was much better than that of the Mo-BVO.

To probe the surface states of the Mo-BVO and the Mo-BVO (C) before and after the stability tests, XPS measurements were also performed. As shown in Figures S9 and S10, C, Bi, V, O and Mo were detected in both samples. Furthermore the actual doping concentrations of Mo in the Mo-BVO and the Mo-BVO (C) were measured at 0.63% and 0.5%, respectively (see Table S2). Although the starting dopant concentration in both the Mo-BVO and the Mo-BVO (C) was the same (1%), more V could volatize during the high-temperature calcination process in the Mo-BVO (C) owing to its higher calcination temperature and longer calcination time. As shown in Figure S9, the Bi 4f and V 3d XPS peaks of the Mo-BVO (C) remained almost unchanged before and after the long-term PEC test. However, in the case of the Mo-BVO, the V 3d XPS peaks clearly became smaller after the PEC test, while the Bi 4f XPS peaks remained unchanged. The initial Bi/V ratio for the Mo-BVO and the Mo-BVO (C) were 1:0.80 and 1:0.9, respectively. Furthemore, after the stability test, the Bi/V ratio for the Mo-BVO and the Mo-BVO (C) became 1: 0.51 and 1: 0.80, respectively. This result accorded with the stability test discussed above, where the decrease of the V 3d XPS peaks for the Mo-BVO was ascribed to the dissociation of V into the electrolyte solution during the long-term PEC test, indicating that the Mo-BVO (C) was more stable than the Mo-BVO during the PEC process. Accordingly, the O 1s XPS peaks corresponding to the surface oxygen species [32,33,34] (hydroxy groups, dangling oxygen) at about 531 eV also greatly changed for the Mo-BVO, while the ratio of surface oxygen species of the Mo-BVO increased from 9.25% to 22.35% before and after the stability test, respectively (see Figure S10E). However, for the Mo-BVO (C), this ratio only slightly increased from 27 % to 30% before and after the stability test. This result was also consistent with the SEM images in Figure 5, where serious photo-corrosion in the Mo-BVO film effectively increased the exposed surface area to electrolyte solution, leading to an increase in the number of surface oxygen species.

According to the experimental results and the analyses discussed above, the Mo-BVO (C) ceramic electrode with higher crystallinity and fewer surface defects was proven to be more stable than the Mo-BVO film consisting of nanoparticles. BiVO_4_ ceramic photoanodes, with their higher crystallinity and fewer surface defects, not only exhibit higher interfacial charge separation efficiency, but are also able to effectively inhibit the dissociation of V from the electrode to the electrolyte solution during the PEC process. As the stability of BiVO_4_ photoanode is vitally important for practical applications, BiVO_4_ ceramics could be potential candidates for the fabrication of highly stable BiVO_4_ photoanodes for PEC water splitting. On the other hand, as it might be difficult to decrease the thickness of BiVO_4_ ceramics to hundreds of nanometers, the PEC stability of BiVO_4_ photoanodes could also be enhanced by improving their crystallinity and reducing the concentration of their surface defects.

## 4. Conclusions

In summary, we fabricated BiVO_4_ ceramics with high density and good crystallinity using the SPS method and investigated their PEC performances and stability. Compared to Mo-BVO films on FTO substrates consisting of BiVO_4_ nanoparticles, the as-prepared Mo-BVO (C) exhibited greatly enhanced PEC stability under simulated solar light irradiation. At 1.23 V vs. RHE, the photocurrent of the Mo-BVO (C) remained almost unchanged at 0.54 mA/cm^2^ for as long as 10 h, while the photocurrent of the Mo-BVO films decreased from 0.66 mA/cm^2^ to 0.11 mA/cm^2^ in 4 h. By comparing the changes to the respective structures, morphologies, compositions, and surface states of the as-prepared Mo-BVO and the Mo-BVO (C) before and after the PEC stability tests, the excellent PEC stability of the Mo-BVO (C) was ascribed to its superior crystallinity and lower concentration of surface defects, which can greatly reduce the dissociation of V from the electrode and prevent photo-corrosion during the PEC process. This study demonstrates a rational design of BiVO_4_ ceramics to improve the stability of the corresponding photoanode, and may be applied to other metal oxide photoanodes in order to maintain their high photostability.

## Data Availability

The data presented in this study are available on request from the corresponding author.

## Supplementary Materials

The following are available online at https://www.mdpi.com/article/10.3390/nano11092404/s1, Figure S1: SEM images of the BiVO_4_ powder precursors to fabricate BVO (C) and Mo-BVO (C), Figure S2: Top-view and cross-sectional SEM images of BVO and Mo-BVO films on FTO substrates, Figure S3: XRD patterns of BVO (C), Mo-BVO (C), BVO and Mo-BVO films on FTO substrates, Figure S4: UV-Vis absorption spectrums of Mo-BVO(C) powder and BVO(C) powder from the Mo-BVO(C) ceramiac and BVO(C) ceramiac ground with a mortar, Figure S5: Photocurrent density versus applied potential (J-V) curves and Photocurrent vs. time (I-T) measured for the BVO(C) and BiVO_4_ film photoanodes with a scan rate of 20 mV/s under AM 1.5G illumination in 0.1 M KPi buffer solution, Figure S6: Nyquist plots of Mo-BVO(C) and Mo-BVO samples, Figure S7: J-V and I-T curves measured for the Mo-BVO and Mo-BVO(C) photoanodes in 0.1M Na_2_SO_3_ KPi solution, Figure S8: IPCEs for Mo-BVO and Mo-BVO(C) measured in the wavelength range from 350 to 550 nm at applied voltage of 1.23 V vs. RHE, Figure S9: XPS patterns of the Mo-BVO(C) electrodes in KPi before and after PEC stability tests, Figure S10: XPS patterns of the Mo-BVO electrodes in KPi before and after PEC stability tests, Table S1. EDS of Mo-BVO and Mo-BVO(C), Table S2: Mo content in Mo-BVO and Mo-BVO(C).
