# Supplementary material for: BiVO4 Ceramic Photoanode with Enhanced Photoelectrochemical Stability
Source: Nanomaterials (Basel). 2021 Sep 15;11(9):2404. doi: 10.3390/nano11092404 (PMC8466786; doi:10.3390/nano11092404)
Supplement: Supplementary file 1 [file nanomaterials-11-02404-s001.zip › nanomaterials-1330150-supplementary.pdf]

nanomaterials

Supplementary Materials

$BiVO_4$ Ceramic Photoanode with enhanced Photoelectrochemical Stability

Liren Zheng [1,2], Minrui Wang [1], Yujie Li [1], Fahao Ma [1], Jiyu Li [1], Weiyi Jiang [1], Mu Liu [1], Hefeng Cheng [1], Zeyan Wang [1,*], Zhaoke Zheng [1], Peng Wang [1], Yuanyuan Liu [1], Ying Dai [3] and Baibiao Huang [1,*]

[1] State Key Laboratory of Crystal Materials, Shandong University, Jinan 250100, China; 201720279@mail.sdu.edu.cn (L.Z.); 201611976@mail.sdu.edu.cn (M.W.); 18306422339@163.com (Y.L.); mafahao@mail.sdu.edu.cn (F.M.); jiyuli1226@gmail.com (J.L.); jiangweiyi1994@mail.sdu.edu.cn (W.J.); liumu@mail.sdu.edu.cn (M.L.); chenghefeng@sdu.edu.cn (H.C.); zkzheng@sdu.edu.cn (Z.Z.); pengwangicm@sdu.edu.cn (P.W.); yyliu@sdu.edu.cn (Y.L.)
[2] School of Physics and Electronic Engineering, TaiShan University, Tai'an 271000, China
[3] School of Physics, Shandong University, Jinan 250100, China; daiy60@sina.com
* Correspondence: wangzeyan@sdu.edu.cn (Z.W.); bbhuang@sdu.edu.cn (B.H.)

1. The Basic Equations of Photoelectrochemistry and Their Calculation Process

The water oxidation of photoanode is a four electron process during PEC water splitting, while the water reduction of photocathode is a two electron reaction. In order to synchronize the reaction, the water oxidation is replaecd with one electron oxidation of Sodium sulfite, so as to achieve an efficient PEC reaction. And ($\mathrm{H_2O}$ oxidation : $2H_2O + 4h^+ \rightarrow 4H^+ + O_2, E_0 = 1.23V_{RHE}$, Na_2SO_3 oxidation : $SO_3^{2-} + h^+ \rightarrow SO_3^-, E^0 = 0.73V_{RHE}$) [1], therefore, with the addition of Na_2SO_3 sacrificial agent, the holes at the interface can be completely reacted, and the injection efficiency can be regarded as 100%.

For characterizing the injection efficiency of Mo- BVO and Mo-BVO(C) photoanodes in KPi, the 0.1 M Na_2SO_3 was added into electrolyte as a hole scavenger, so the injection efficiency (η_i) was characterized by the equation (1) [2]:

$$\eta_\mathrm{i} = \frac{J_{ph}^{H_2O}}{J_{ph}^{Na_2SO_3}}, \quad (1)$$

According to the formula: $\eta_\mathrm{ph} = \eta_\mathrm{b} \times \eta_\mathrm{i} \times J_\mathrm{abc}$. So the bulk carrier separation efficiency (η_b) was calculated by the equation (2) [2,3]:

$$\eta_\mathrm{b} = \frac{J_{ph}^{Na_2SO_3}}{J_{abc}}, \quad (2)$$

where J_{abc} is the photocurrent density which it's assumed that the photoelectric conversion efficiency of the absorbed sunlight is 100%.

And the J_{abc} can be calculated by following equation (3) [4]:

$$J_{abc}=\frac{1}{E_g}\int_0^{\lambda_i} A_\lambda \Phi_\lambda d\lambda = \int_{\lambda_1}^{\lambda_2} \frac{\lambda(nm)}{1239.8(V\bullet nm)} \times \frac{P_{abc}(\lambda)}{10}\left(\frac{mW}{cm^2\bullet nm}\right) d\lambda, \quad (3)$$

where A_λ is the absorbance and Φ_λ is the photon flux of the AM 1.5G solar spectrum. $P_{abs}(\lambda)$ [mW/(cm²·nm)] is the photon flux of AM 1.5G solar spectrum actually absorbed by the photoelectrode.

The J_{abc} of Mo-BVO and Mo-BVO(C) were estimated by integrating the overlap region of the AM 1.5G sunlight spectrum and the UV-Vis absorption spectra of Mo-BVO and Mo-BVO(C), respectively. And then divided by the bandgap of the Mo-BVO and Mo-BVO(C) semiconductor ($E_{g,\text{ Mo-BVO}} = 2.47eV$, $E_{g,\text{ Mo-BVO(C)}} = 2.40eV$). So the J_{abc} values of Mo-BVO BVO and Mo-BVO(C) are calculated to be 3.50 and 3.90 mA cm^{-2}, respectively. So we can calculate the internal separation efficiency of the BVO and Mo-BVO(C) photoanodes. In the equation (3), λ and P_{abc} are variables. Therefore, it is necessary to integrate the product of these two quantities. The integration result of the product of the two variables is the common area between the blue line and the red line (or black line) and the abscissa (wavelength, λ) in the range from 300 nm to 510 nm, See the Fgure below. Then the area results of film and ceramic can be calculated, respectively. Then the two theoretical photocurrents are calculated by using the equation (3). The wavelength range corresponding to the theoretical photocurrent of the thin film electrode is calculated from 300 nm to 500 nm. That of the ceramic electrode is calculated from 300 nm to 517 nm, see the figure below:

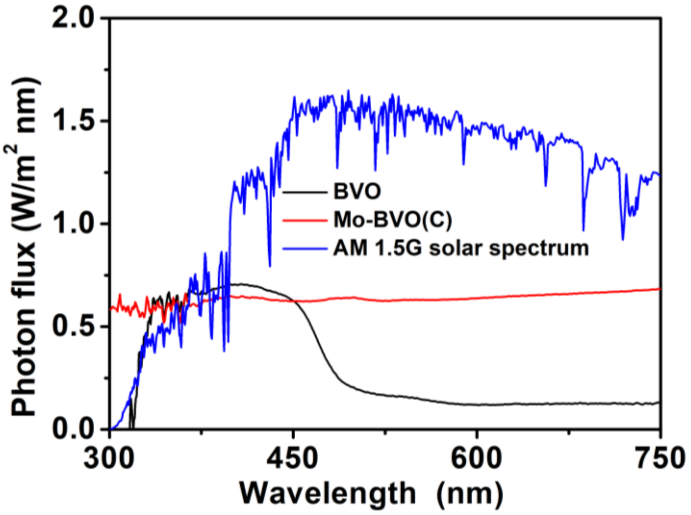

Comparison of AM 1.5G sunlight spectrum and UV-Vis absorption spectrums of BVO and Mo-BVO(C) photoanodes.

Then the area results of film and ceramic can be calculated, respectively. Then the two theoretical photocurrents are calculated by using the equation (3). The wavelength range corresponding to the theoretical photocurrent of the thin film electrode is calculated from 300 nm to 500 nm. That of the ceramic electrode is calculated from 300 nm to 517 nm (Figure S5).

The band gap energy was estimated by using the equation (4) [2,3]:

$$(\alpha h\nu)^n = h\nu - E_g, \tag{4}$$

where α is the absorption coefficient, hν is the photon energy (h is Plank's constant, ν is the frequency), n is a constant with a value of 2 for direct-bandgap semiconductor, and the E_g is the bandgap energy.

The incident-photon-to-current-conversion efficiency (IPCE) measurements were performed by measuring the photocurrent density under monochromated light irradiation with a 500 W Xe arc lamp coupled into a grating monochromator, which were then calculated following the equation (5) [5]:

$$IPCE = \frac{1239.8(V\bullet \mathrm{nm}) \times \left| I_{ph}\left(mA\bullet cm^{-2}\right)\right|}{\lambda(nm) \times P_{mono}\left(mW\bullet cm^{-2}\right)} \times 100\%, \tag{5}$$

where I_{ph} is the photocurrent density, λ the incident light wavelength, and P_{mono} is the calibrated and monochromated illumination power intensity. The illumination intensities of the monochromatic light were measured with a PM 100A Optical Power Meter (ThorLabs).

2. Some Experimental Data

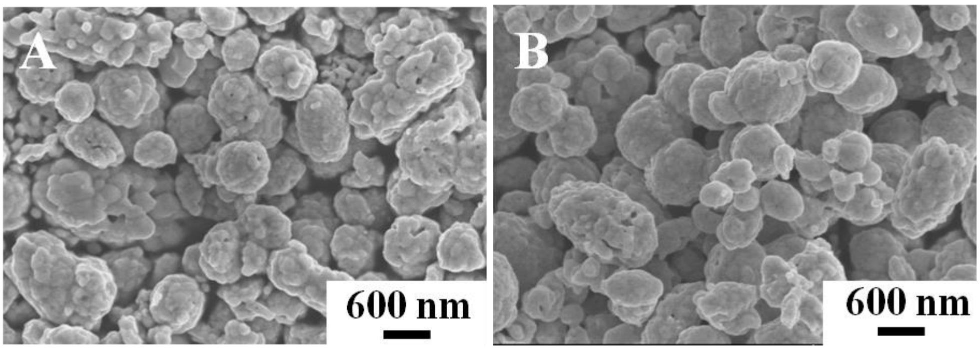

Figure S1. (**A**) and (**B**) SEM images of the $BiVO_4$ powder precursors to fabricate BVO (C) and Mo-BVO (C).

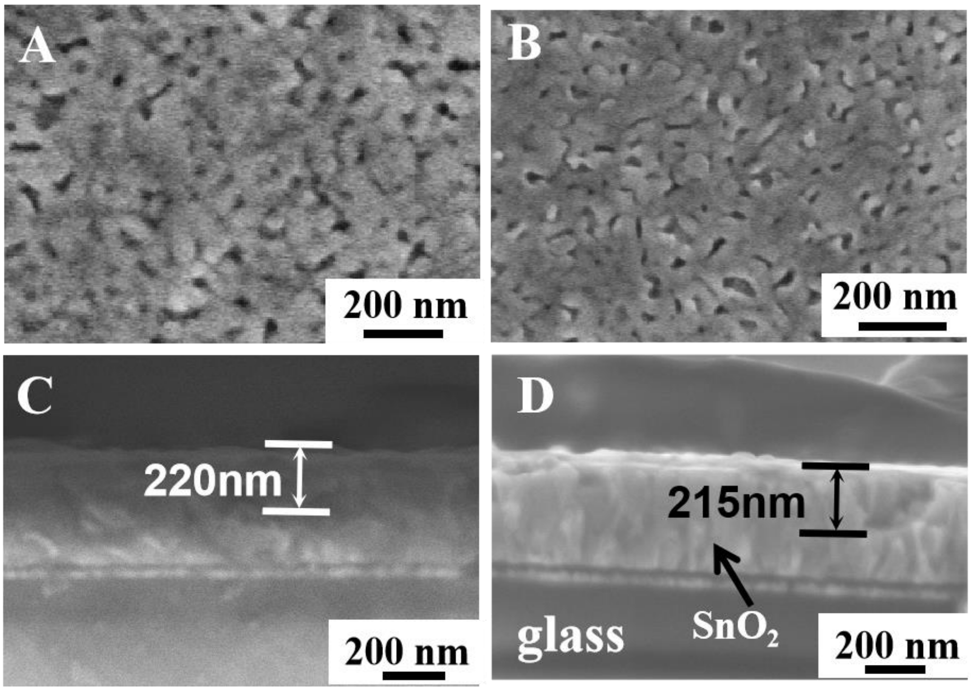

Figure S2. (**A**) and (**B**) Top-view and cross-sectional SEM images of BVO and Mo-BVO films on FTO substrates.(**C**) and (**D**) Cross-sectional images of the as-prepared BVO and Mo-BVO.

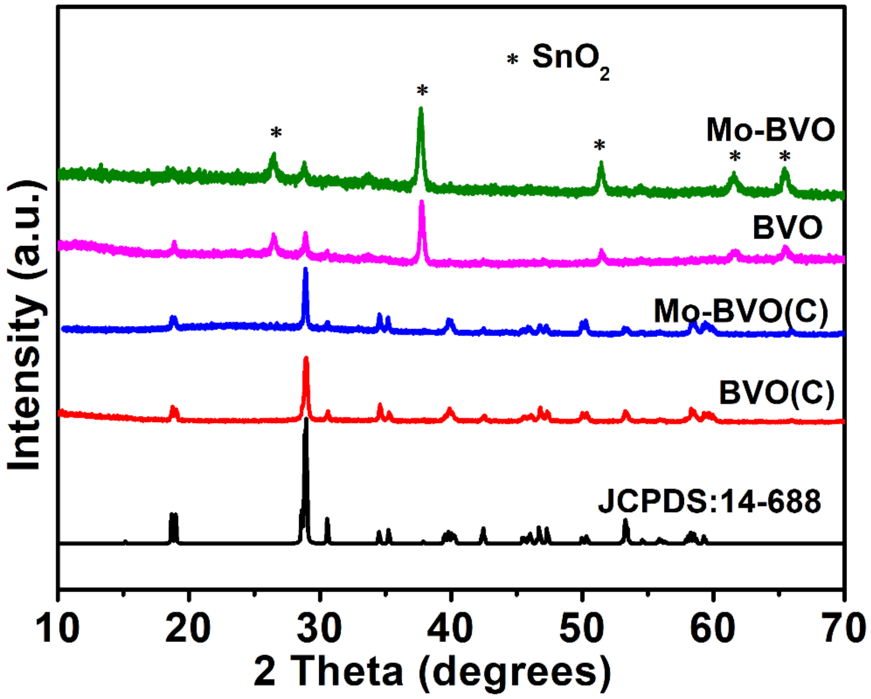

Figure S3. XRD patterns of BVO (C), Mo-BVO (C) and XRD patterns of BVO and Mo-BVO films on FTO substrates.

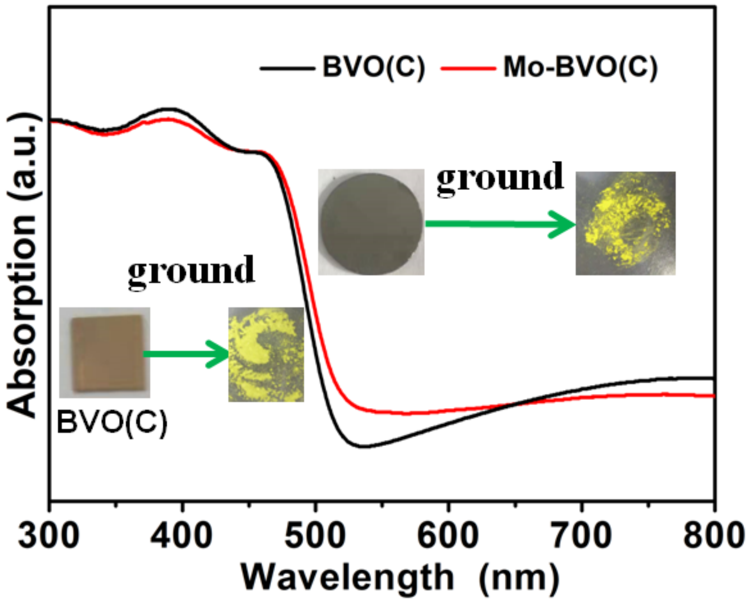

Figure S4. UV-Vis absorption spectrums of Mo-BVO(C) powder and BVO(C) powder from the Mo-BVO(C) ceramiac and BVO(C) ceramiac ground with a mortar. Photos before and after the Mo-BVO ceramic grinding are shown as insets.

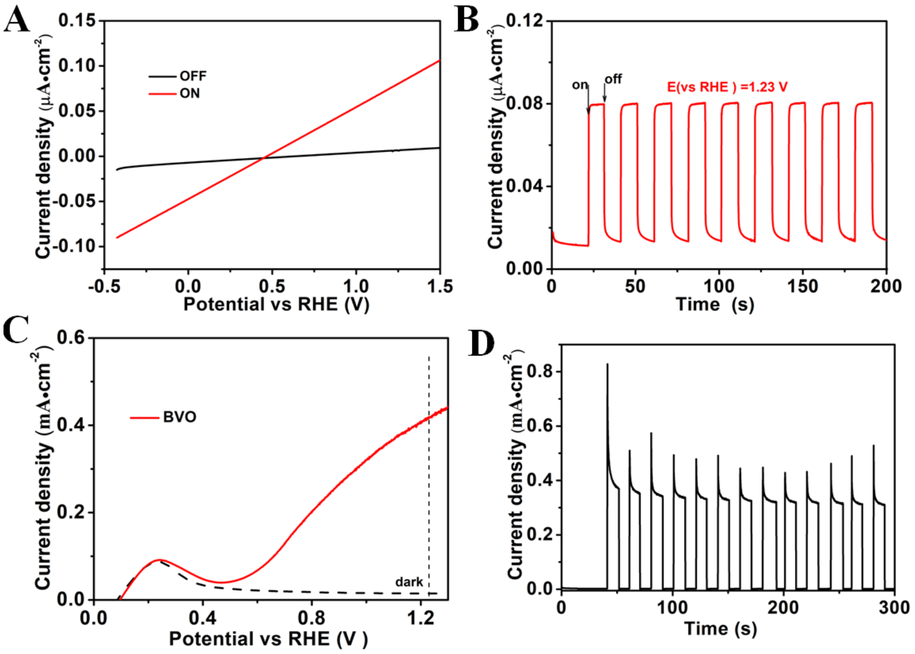

Figure S5. (**A**) Photocurrent density versus applied potential (J-V) curves measured for the BVO(C) photoanodes with a scan rate of 20 mV/s under AM 1.5G illumination in 0.1 M KPi buffer solution. (**B**) Photocurrent vs. time (I-T) measurements performed with chopped the light (AM 1.5G) at 1.23 V (vs RHE) in phosphate buffer (pH = 7) solution. (inset shows the digital photos of BVO(C)). (**C**) Photocurrent density versus applied potential (J-V) curves measured for the $BiVO_4$ film photoanode with a scan rate of 20 mV/s under AM 1.5G illumination in 0.1 M KPi buffer solution.(**D**) Photocurrent vs. time (I-T) measurements performed with chopped the light (AM 1.5G) at 1.23 V (vs RHE) in phosphate buffer (pH=7) solution.

Table 1. EDS of Mo-BVO and Mo-BVO(C).

	Element (Atomic%) (Before)				Element (Atomic%) (After)			
	**O**	**Bi**	**V**	**Mo**	**O**	**Bi**	**V**	**Mo**
Mo-BVO	91.06	4.79	4.15	0	95.29	2.76	1.95	0
Mo-BVO(C)	62.32	20.84	16.84	0	61.24	22.36	16.41	0

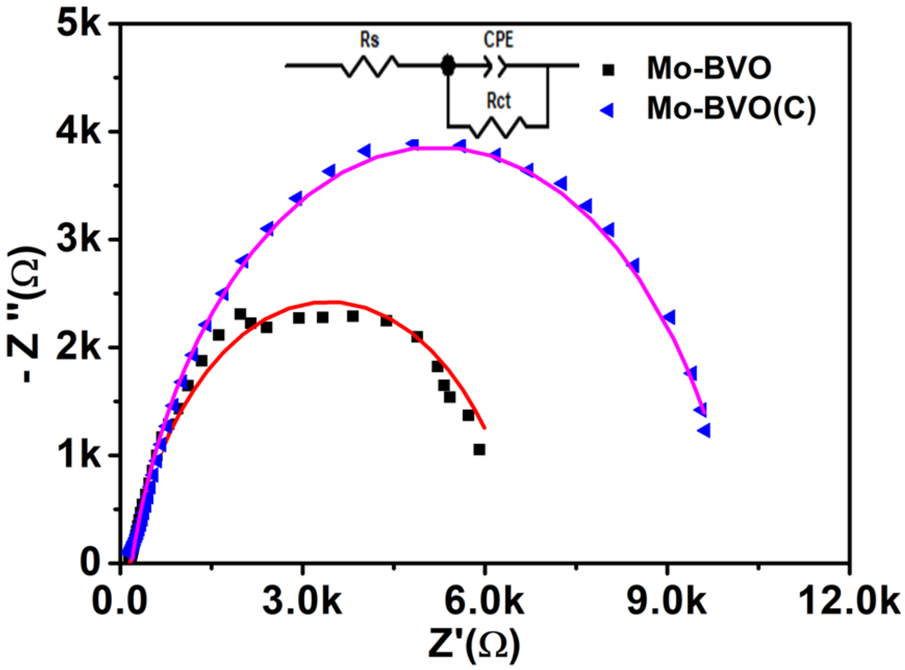

Figure S6. Nyquist plots of Mo-BVO(C) and Mo-BVO samples. The equivalent circuit from their fitted data is shown as inset.

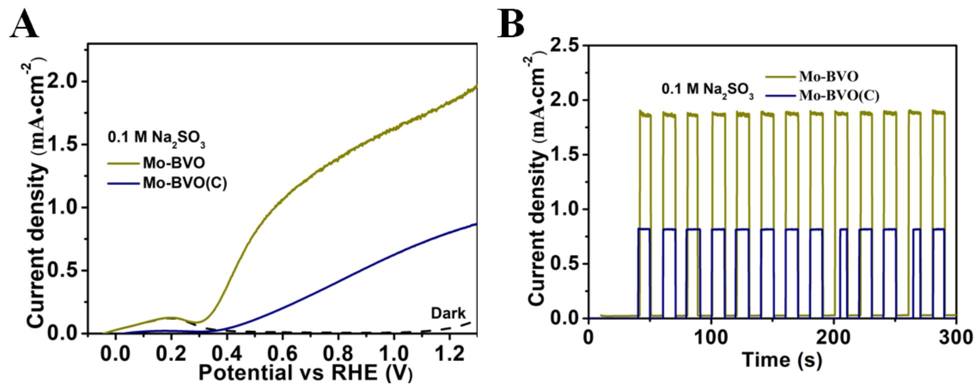

Figure S7. (**A**) J-V curves measured for the Mo-BVO and Mo-BVO(C) photoanodes in 0.1M Na_2SO_3 KPi solution. (**B**) I-T measurements performed with chopped the light (AM 1.5G) at 1.23 V (vs RHE) in 0.1M Na_2SO_3 KPi solution.

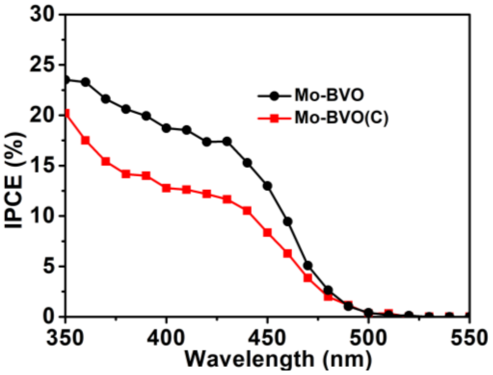

Figure S8. IPCEs for Mo-BVO and Mo-BVO(C) measured in the wavelength range from 350 to 550 nm at applied voltage of 1.23 V vs RHE.

Table S2. Mo content in Mo-BVO and Mo-BVO(C).

Dopant	sample	Starting (%)	Final(%)
Mo	Mo-BVO	1	0.63
	Mo-BVO(C)	1	0.50

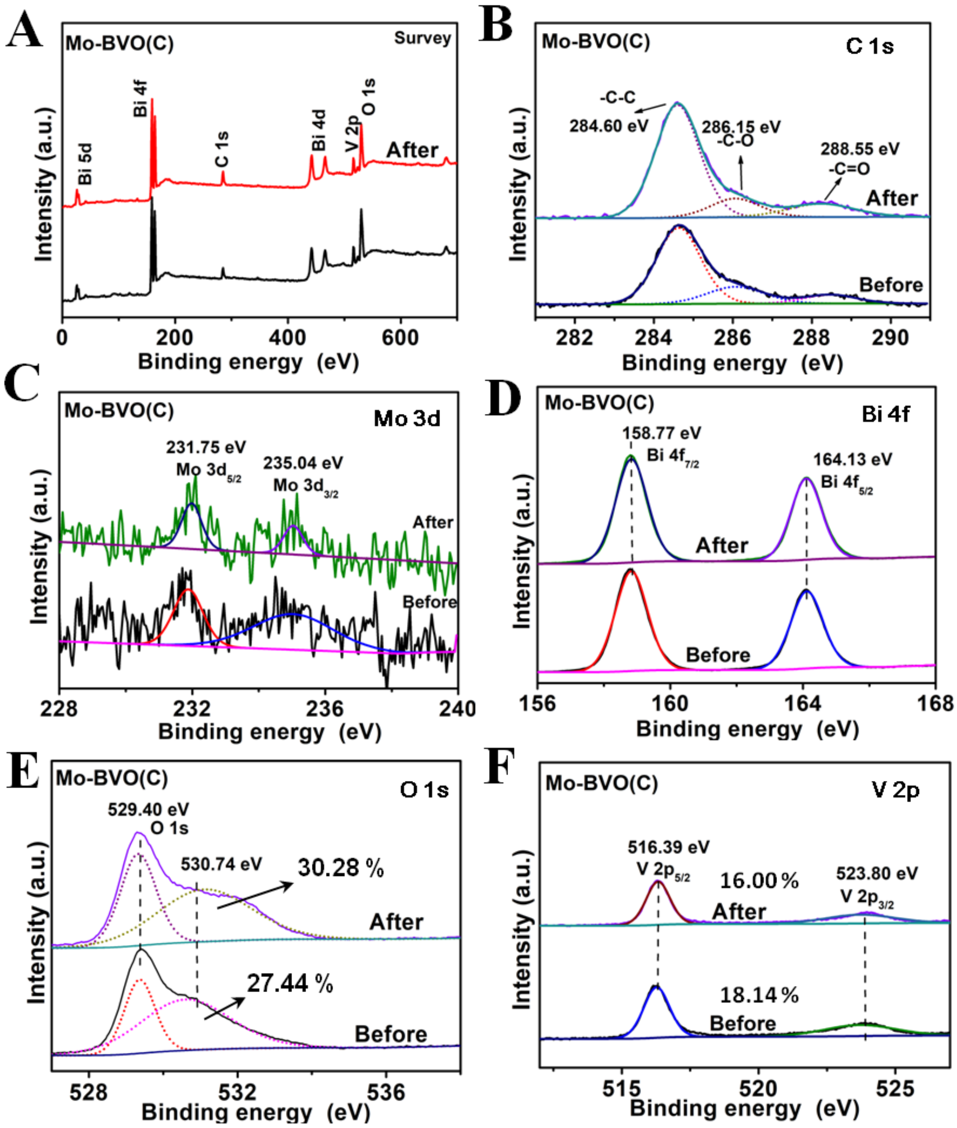

Figure S9. XPS patterns of the Mo-BVO(C) electrodes in KPi before and after PEC stability tests. (**A**) Survey. (**B**) C 1s peaks. (**C**) Mo 3d peaks. (**D**) Bi 4f peaks. (**E**) O1s peaks. (**F**) V 2p peaks.

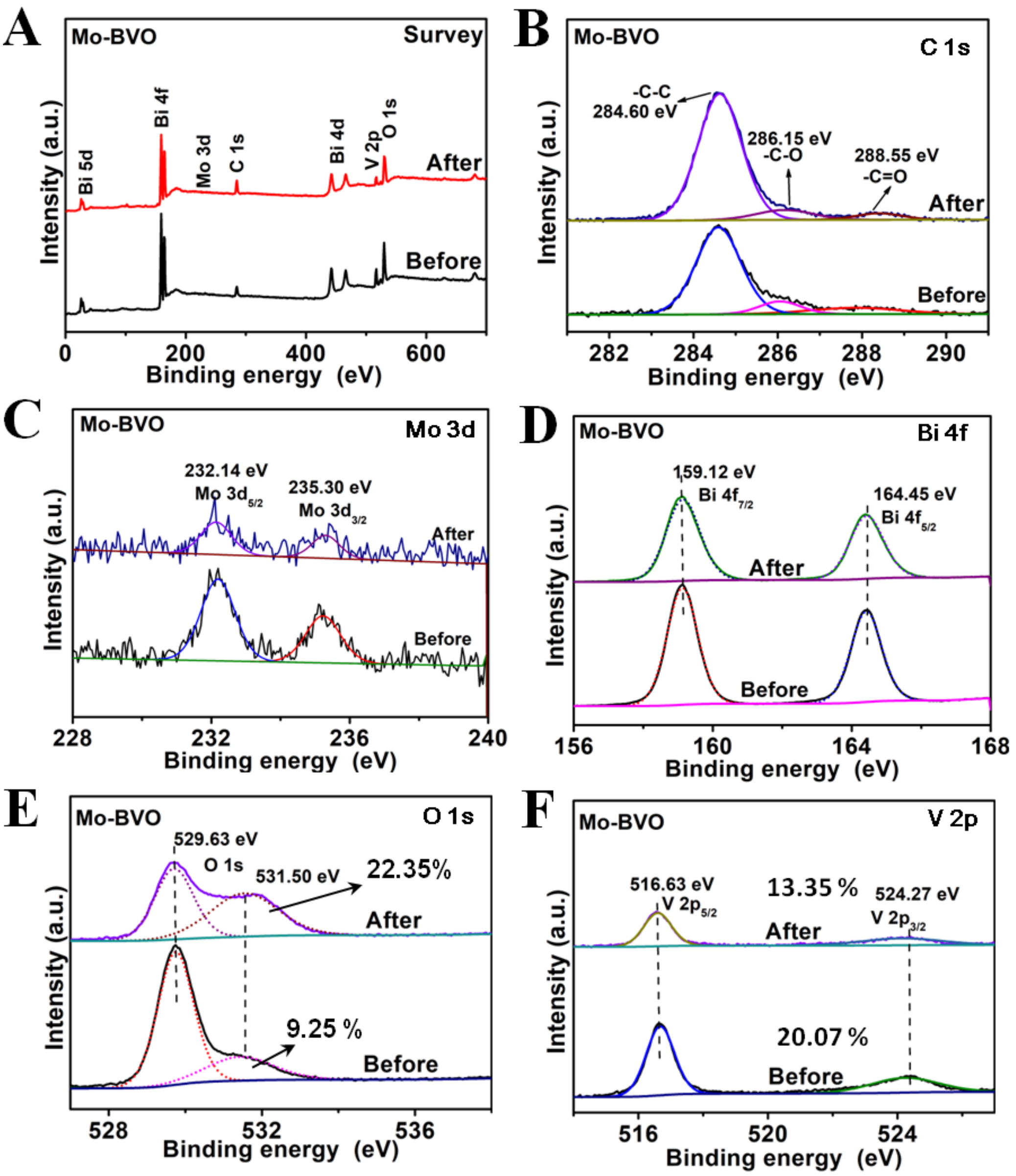

Figure S10. XPS patterns of the Mo-BVO electrodes in KPi before and after PEC stability tests. (**A**) Survey. (**B**) C 1s peaks. (**C**) Mo 3d peaks. (**D**) Bi 4f peaks. (**E**) O1s peaks. (**F**) V 2p peaks.

References

1. Kim, J. H.; Lee, J. S.; Elaborately Modified $BiVO_4$ Photoanodes for Solar Water Splitting. *Adv. Mater.* **2019**, *31*, 1806938
2. Murphy, A. B.; Band-gap determination from diffuse reflectance measurements of semiconductor films, and application to photoelectrochemical water-splitting. *Sol. Energy Mater. Sol. Cells.* **2007**, *91*, 1326–1337.
3. Willardson, R. K.; Beers (Eds.), A. C. Optical Properties of III-V Compounds, Semiconductors and Semimetals, vol. 3, Academic Press, New York **1967** , pp. 153-258.
4. Dotan, H.; Sivula, K.; Grätzel, M.; Rothschild, A.; Warren, S. C Probing the Photoelectrochemical Properties of Hematite (α-Fe_2O_3) Electrodes Using Hydrogen Peroxide as a Hole Scavenger. *Energy Env. Sci*. **2011**,*4* , 958–964.
5. Zhang, B.; Zhang, H. P.; Wang, Z. Y.; Zhang, X. Y.; Qin, X. Y.; Dai, Y.; Liu, Y. Y.; Wang, P.; Li, Y. J.; Huang, B. B. Doping Strategy to Promote the Charge Separation in $BiVO_4$ Photoanodes. *Appl. Catal. B Environ*. **2017**, *211*,258–265.
